# The Usefulness of Assessing and Identifying Workers’ Temperaments and Their Effects on Occupational Stress in the Workplace

**DOI:** 10.1371/journal.pone.0156339

**Published:** 2016-05-26

**Authors:** Yasuhiko Deguchi, Shinichi Iwasaki, Akihito Konishi, Hideyuki Ishimoto, Koichiro Ogawa, Yuichi Fukuda, Tomoko Nitta, Koki Inoue

**Affiliations:** Department of Neuropsychiatry, Graduate School of Medicine, Osaka City University, Osaka, Japan; University of Illinois at Chicago, UNITED STATES

## Abstract

The relationship between temperaments and mental disorders has been reported in previous studies, but there has been little attention to temperaments in the occupational safety and health research. The aim of this study was to clarify the effects of temperaments on occupational stress among local government employees. The subjects were 145 Japanese daytime workers in local government. Temperaments were assessed by the Temperament Evaluation of Memphis, Pisa, Paris, and San Diego-Auto questionnaire (TEMPS-A). Occupational stress was assessed using the Generic Job Stress Questionnaire (GJSQ). Hierarchical multiple linear regression analysis was used. Hyperthymic temperament predicted a higher level of job control, and a lower level of role ambiguity and job future ambiguity. Irritable temperament predicted a lower level of social support from supervisors and a higher level of role conflict, variance in workload and intragroup conflict. Anxious temperament predicted a lower level of social support from coworkers and a higher level of job future ambiguity. The sample size was small. Only Japanese local government employees were surveyed. Hyperthymic temperament played a protective role, and irritable, anxious temperament played a vulnerable role against one’s own occupational stress and recognizing the roles they play in work life would lead to self-insight. Additionally, recognition of the temperaments and temperament-related stressors by one’s supervisors or coworkers would facilitate provision of social support.

## Introduction

Temperament has been defined as heritable personality factors remaining stable over time and establishing a person’s baseline level of mood, reactivity and energy [1]. Temperament has been associated with genetic factors that define personality, and personality has been argued to be formed through the process of development and the experiences of daily life [2]. References to an optimum mixture of human attributes have been identified as far back as ancient Greece. In the early 20^th^ century, Kraepelin proposed four fundamental states (depressive, cyclothymic, manic and irritable) that correspond to subclinical manifestations of manic-depressive illness. On the basis of these theories, Akiskal formulated the modern concept of five affective temperaments, adding the anxious type to the initial four types, and suggested that affective temperaments were the subclinical manifestations or phenotypes of mood disorders, representing one end of the continuum of affective illness [3, 4].

Many studies have reported relationships between temperaments and mental problems, e.g., suicide [5, 6], mental status in non-clinical populations [7], depressive symptoms [8–10], mood disorders [11–15], anxiety disorders [16], alcoholic abuse or dependence [17, 18], substance abuse [19, 20], and smoking maintenance [21].

Two of the most commonly used assessments of temperament are the Temperament Evaluation of Memphis, Pisa, Paris, and San Diego-Auto (TEMPS-A) and the Temperament and Character Inventory (TCI). Akiskal developed the TEMPS-A questionnaire for temperament research and clinical purposes [3, 22]. Unlike personality, temperaments evaluated by TEMPS-A did not change substantially over 6 years [23]. The TCI was developed by Cloninger and colleagues [24]. The relationships between temperament assessed by TCI and major depression [25], bipolar disorder [26], suicidal behavior [27] and other conditions have been reported. Concurrent validity of TEMPS-A with TCI has been documented [22]. We used TEMPS-A in this study because, at 110 items, it has the advantage of brevity over TCI.

Temperament is thought to include the inherent biases in the way that persons view and act, and to be influenced by environmental factors in the formation of personality [28]. In the workplace, depressive temperament has been reported to be a kind of work-oriented personality [29] and hyperthymic temperament has been reported to be a kind of hyper-adapted personality [30]. Different workers in the same occupational environment might have different occupational stress, and such differences might derive from their different individual temperaments. Therefore, recognizing temperaments and their effects on occupational stress would be useful for addressing mental health problems. However, temperaments have received little attention in occupational safety and health research. The few studies that exist (among IT service company employees and nurses) have produced conflicting results, especially with regard to the relationships between hyperthymic, irritable temperament and occupational stress [30–32]. Therefore, research with other kinds of occupations is needed before firm conclusions can be drawn.

We hypothesized that temperaments can play a protective or vulnerable role against occupational stress, and that different temperaments can produce higher-than-expected or lower-than-expected occupational stress. The aim of this study was to clarify the effects of temperaments on occupational stress among local government employees.

## Materials and Methods

### Subjects

We distributed the self-administered, anonymous questionnaires to 172 Japanese day-shift workers in local government between August and September 2014. One hundred and forty-five workers completed the questionnaire (response rate 84.3%). All participants gave their informed consent to participate as volunteers, and understood that there was no penalty for choosing not to participate.

### Ethics statement

The study design was approved by the Human Subjects Review Committee at Osaka City University (authorization number: 2969). All data were stored only in our database, and the employer did not have access to the data or know who had participated in the study.

### Measures

#### Demographic and work-related variables

Demographic variables included age and gender. Work-related variables included service years, position classification (non-manager, manager), occupation (clerical, professional), and type of employment (regular, temporary).

#### Measures of temperament

Temperaments were assessed by the full version of the TEMPS-A developed by Akiskal et al. [3, 22]. The reliability and validity of the Japanese version have been established [13]. The TEMPS-A assesses emotional, cognitive, psychomotor, interpersonal and vegetative (such as sleep, sexual desire) dimensions, which are argued to play a vulnerable or adaptive role in an evolutionary context and with regard to predisposition to major mood disorders [3]. The TEMPS-A is a true-false questionnaire and measures affective temperaments defining the bipolar spectrum. The instrument’s 110 items are divided into five types of temperaments: depressive, cyclothymic, hyperthymic, irritable, and anxious. Higher scores suggest greater magnitude of the temperaments.

#### Measures of occupational stress

Occupational stress was assessed using the Generic Job Stress Questionnaire (GJSQ) developed by the National Institute for Occupational Safety and Health (NIOSH) [33]. The Japanese version of the GJSQ has demonstrated sufficient reliability and validity [34, 35]. We chose the GJSQ because it can assess multilateral aspects of occupational stress, including stress reactions, at the group and individual levels. The original authors of the GJSQ permit the use of their independent subscales for assessing occupational stress [33], and we focused on 7 subscales (68 items) to assess occupational stress: role conflict, role ambiguity, quantitative workload, variance in workload, intragroup conflict, job control, and job future ambiguity. We also chose two social support subscales (from supervisors, from coworkers) that function as buffer factors, according to the results of a previous study [30, 36, 37]. For the job control and social support items, item descriptions are positively oriented, so that higher scores indicate lower stress. In contrast, all other items are negatively oriented, so that higher scores indicate greater stress.

Role conflict measures how often workers experience role conflict with each other. Role ambiguity measures how clearly the worker understands what is expected of him or her for adequately performing a role or task. Quantitative workload refers to the amount of work a person has to deal with on a daily basis. Variance in workload measures the extent to which the work tasks demand speediness and concentration, and are generally difficult to handle. Intragroup conflict measures the harmony, conflict or dialogue discrepancy in the group. Job control refers to how much the individual feels that his or her tasks, workplace setting, and decisions at work are controllable. Job future ambiguity refers to how much the position advancement, skills and responsibilities on work are perspective. Social support from supervisors and coworkers measures existence of avenues for acquiring social support during work time.

### Statistical analyses

The correlation between TEMPS-A temperament scores and occupational stress was analyzed by Spearman’s correlation. As gender, age and work-related variables are related to occupational stress, we performed hierarchical multiple linear regression analysis to determine whether temperament scores of TEMPS-A explained a significant variance to each of the nine occupational stress. Gender, age and work-related variables were entered in step 1, and temperament variables were entered in step 2, with controls for the variables from step 1. Differences were considered significant at *p* < 0.05. All statistical analyses were performed using SPSS version 21.0 software (SPSS Inc., Chicago, IL).

## Results

### Subjects’ characteristics

Table 1 shows the subjects’ characteristics, TEMPS-A temperament scores, and mean GJSQ scores. The mean age and service years of the study population were 40.8 ± 12.1 and 13.3 ± 12.1 years, respectively. The sample comprised 54 (37.2%) men and 91 (62.8%) women, 101 non-managers (69.7%) and 44 (30.3%) managers, 107 (73.8%) clerical and 38 (26.2%) professional workers, and 110 (75.9%) regular and 35 (24.1%) temporary workers.

**Table 1:** Demographic variables, GJSQ scores and TEMPS-A scores.

	N	(%)
Gender		
Male	54	(37.2)
Female	91	(62.8)
Position classification		
Non-manager	101	(69.7)
Manager	44	(30.3)
Occupation		
Clerical	107	(73.8)
Professional	38	(26.2)
Type of employment		
Regular	110	(75.9)
Temporary	35	(24.1)
	Range	Mean±SD
Age		40.8±12.1
Service years		13.3±12.1
GJSQ scores		
Role conflict	(8–56)	25.7±8.3
Role ambiguity	(6–42)	18.6±5.1
Quantitative workload	(11–55)	34.4±7.3
Variance in workload	(3–15)	7.6±2.7
Intragroup conflict	(8–40)	19.0±5.4
Job control	(16–80)	41.7±11.7
Job future ambiguity	(4–20)	14.9±4.2
Supervisors support	(4–20)	15.5±3.2
Coworkers support	(4–20)	16.3±3.1
Temperaments		
Depressive	(0–21)	8.1±3.5
Cyclothymic	(0–21)	4.2±3.9
Hyperthymic	(0–21)	5.2±4.2
Irritable	(0-Male 21, Female 22)	2.9±3.1
Anxious	(0–26)	6.0±5.4

GJSQ: Generic Job Stress Questionnaire

### Correlations between temperaments and occupational stress

Table 2 shows the Spearman’s correlations between the five temperaments and occupational stressors according to the GJSQ. The results were interpreted based on Guilford’s rule of thumb [38]. Cyclothymic temperament score was weakly correlated with role conflict, role ambiguity, intragroup conflict, job future ambiguity and social support from coworkers. Hyperthymic temperament score was weakly correlated with role ambiguity and job control. Irritable temperament score was moderately correlated with role conflict, weakly correlated with role ambiguity, quantitative workload, variance in workload, intragroup conflict, job future ambiguity, social support from supervisors and social support from coworkers. Anxious temperament score was weakly correlated with role conflict, job future ambiguity, social support from supervisors and social support from coworkers. Depressive temperament score was not significantly correlated with any of the occupational stressors.

**Table 2:** Correlation between temperaments and occupational stress.

	Role conflict	Role ambiguity	Quantitative workload	Variance in workload	Intragroup conflict	Job control	Job future ambiguity	Supervisors support	Coworkers support
Depressive	0.13	0.114	0.114	0.041	0.026	-0.177	0.163	-0.169	-0.124
Cyclothymic	0.223**	0.226**	0.082	0.093	0.237**	-0.093	0.226**	-0.148	-0.246**
Hyperthymic	-0.034	-0.282**	-0.031	0.082	0.105	0.23**	-0.147	0.042	0.021
Irritable	0.4***	0.203*	0.208*	0.243**	0.328***	-0.016	0.226**	-0.233**	-0.261**
Anxious	0.269**	0.137	0.155	0.115	0.142	-0.103	0.326***	-0.183*	-0.285**

* *p* < 0.05

** *p* < 0.01

*** *p* < 0.001

### Hierarchical multiple linear regression analysis examining the associations between occupational stress and temperaments

Table 3 shows the results of the hierarchical multiple linear regression analysis of scores for each GJSQ subscale and the TEMPS-A temperament scores. In step 1 of role conflict, gender was a significant predictor. In step 2, temperament accounted for an additional 17.2% of the variance (*F* = 6.14, *p* < 0.001): irritable temperament predicted a higher level of role conflict and age predicted a lower level. In step 1 of role ambiguity, age was a significant predictor. In step 2, temperament accounted for an additional 16% of the variance (*F* = 5.58, *p* < 0.001): hyperthymic temperament predicted a lower level of role ambiguity. In step 1 of quantitative workload, position classification was a significant predictor. In step 2, temperament accounted for an additional 6.3% of the variance (*F* = 1.98, n.s.): no factors predicted a significant level of quantitative workload. In step 1 of variance in workload, position classification was a significant predictor. In step 2, temperament accounted for an additional 9.7% of the variance (*F* = 3.46, *p* < 0.01): irritable temperament and position classification predicted a higher level of variance in workload. In step 1 of intragroup conflict, there was no significant predictor. In step 2, temperament accounted for an additional 14.3% of the variance (*F* = 4.60, *p* < 0.01): irritable temperament predicted a higher level of intragroup conflict. In step 1 of job control, position classification was a significant predictor. In step 2, temperament variables accounted for an additional 10.4% of the variance (*F* = 3.51, *p* < 0.01): hyperthymic temperament and position classification both predicted a higher level of job control. In step 1 of job future ambiguity, age was a significant predictor. In step 2, temperament accounted for an additional 14.1% of the variance (*F* = 4.86, *p* < 0.001): hyperthymic temperament predicted a lower level and anxious temperament predicted a higher level of job future ambiguity. In step 1 of social support from the supervisor, there was no significant predictor. In step 2, temperament accounted for an additional 11.6% of the variance (*F* = 3.58, *p* < 0.01): irritable temperament predicted a lower level of social support from a supervisor. In step 1 of social support from coworkers, there was no significant predictor. In step 2, temperament accounted for an additional 10.2% of the variance (*F* = 3.23, *p* < 0.05): anxious temperament predicted a lower level of social support from coworkers.

**Table 3:** Hierarchical multiple linear regression analysis, the temperament effects for occupational stress.

	Role conflict		Role ambiguity		Quantitative workload		Variance in workload		Intragroup conflict	
	Step 1	Step2	Step 1	Step2	Step 1	Step2	Step 1	Step2	Step 1	Step2
	β	β	β	β	β	β	β	β	β	β
Gender	-0.189*	-0.251**	-0.041	-0.063	-0.012	-0.039	-0.07	-0.099	0.05	0.009
Age	-0.041	-0.026	-0.257**	-0.174	-0.087	-0.082	-0.011	-0.008	0.009	0.035
Position classification	0.071	-0.044	0.033	0.022	0.211*	0.174	0.297**	0.283**	0.121	0.091
Occupation	0.088	-0.14	0.022	0.05	0.097	0.131	0.027	0.075	0.026	0.075
Type of employment	-0.129	-0.124	-0.036	0.012	-0.099	-0.103	-0.142	-0.149	0.084	0.075
Temperaments										
Depressive		-0.109		-0.043		0.048		0.01		-0.065
Cyclothymic		0.021		0.222		-0.144		0.01		-0.023
Hyperthymic		0.02		-0.316***		0.001		0.069		0.046
Irritable		0.308**		0.2		0.18		0.267*		0.372***
Anxious		0.183		-0.052		0.172		0.038		0.057
R	0.278	0.499	0.262	0.478	0.283	0.379	0.385	0.495	0.148	0.406
R^2^	0.077	0.249	0.068	0.229	0.08	0.143	0.148	0.245	0.022	0.165
R^2^ Change score	0.077	0.172	0.068	0.16	0.08	0.063	0.148	0.097	0.022	0.143
F	2.32*	6.142***	2.043	5.577***	2.421*	1.982	4.835***	3.455**	0.62	4.597**
	Job control		Job future ambiguity		Supervisors support		Coworkers support			
	Step 1	Step2	Step 1	Step2	Step 1	Step2	Step 1	Step2		
	β	β	β	β	β	β	β	β		
Gender	-0.043	-0.039	0.035	-0.018	0.112	0.151	-0.012	0.042		
Age	0.147	0.128	-0.183*	-0.137	-0.007	-0.022	-0.151	-0.142		
Position classification	0.193*	0.205*	-0.092	-0.123	-0.02	0.006	-0.043	-0.028		
Occupation	0.011	-0.001	-0.122	-0.094	0.034	-0.016	-0.036	-0.078		
Type of employment	-0.099	-0.136	0.043	0.082	-0.004	-0.013	0.149	0.137		
Temperaments										
Depressive		-0.123		-0.102		-0.023		0.046		
Cyclothymic		-0.014		0.054		0.01		-0.017		
Hyperthymic		0.230**		-0.217*		0.035		-0.008		
Irritable		0.081		0.141		-0.245*		-0.134		
Anxious		-0.117		0.229*		-0.135		-0.236*		
R	0.319	0.454	0.281	0.47	0.127	0.363	0.229	0.393		
R^2^	0.102	0.206	0.079	0.22	0.016	0.132	0.053	0.155		
R^2^ Change score	0.102	0.104	0.079	0.141	0.016	0.116	0.053	0.102		
F	3.156*	3.513**	2.384*	4.863***	0.455	3.577**	1.545	3.233*		

* *p* < 0.05

** *p* < 0.01

*** *p* < 0.001

Step 1: adjusted for gender, age, and work-related variables, Step 2: adjusted for 5 temperaments, gender, age, and work-related variables.

## Discussion

This study identified the temperaments’ effects on occupational stress using TEMPS-A and GJSQ among Japanese local government employees. We discuss here the five temperaments’ and the demographic variables’ effects on occupational stress, and suggest prevention strategies for work-related mental health problems.

### Temperaments’ effects on occupational stress

#### Hyperthymic temperament and occupational stress

Hyperthymic temperament has been associated with many positive traits: being upbeat, fun-loving, outgoing, jocular, optimistic and confident; full of ideas and eloquent; active, a short-sleeper, but tireless; and preference for leadership; however the temperament is also associated with single-mindedness, risk-taking, and a low likelihood of admitting his or her meddlesome nature [3]. Hyperthymic temperament could be a protective factor against anxiety, bipolar, substance abuse, and impulse control disorders [16] and against suicide among patients with unipolar and bipolar disorder [5].

Workers with hyperthymic temperament may have positive cognitions that inoculate them against occupational stress and work environments that are negative and uncontrollable, and a positive working environment for workers with hyperthymic temperament (such as a pay-per-performance system) might have been fostered by change in society. A previous study reported that hyperthymic temperament carries vulnerability to changes in workload and quantitative workload [30]. Hyperthymic temperament has also been positively associated with happiness, fullness of thought, doing many and exciting things, exuberance, grandiosity, perceiving daily life as positive, a preference for being with others in daily life [39], and feeling calm and peaceful after a stressor [40]. In sum, hyperthymic temperament played a protective role against the workers’ own occupational stress. But the characteristics of being the “single-minded, risk-taker, and unlikely to admit to his/her meddlesome nature” [3] may have negative effects on surrounding coworkers, and these effects should be studied in the future.

#### Irritable temperament and occupational stress

Irritable temperament emerged with two characteristics—skeptical and critical—that might be considered intellectual virtues; otherwise, this temperament has the darkest nature of all: grouchy, complaining, dissatisfied, anger- and violence-prone, and sexually jealous [3]. Workers with irritable temperament may have vulnerability to changes in the work environment and in job relations, and therefore they tended especially to have aggression or stressful feelings for supervisors. Sakai et al. reported that irritable temperament has vulnerability to role ambiguity [30]. Irritable temperament has also been positively associated with negativity, pessimism, boredom, restlessness and reduced desire for social contact, and negatively associated with a preference for being with others in daily life [39] and with feeling well and peaceful [40]. No vulnerability to role ambiguity was found in this study. This may be because workers in our study had had several years to clarify their occupational roles and therefore may have become able to utilize their skills adequately. Another explanation may be the use of a different occupation studied previously. In any case, irritable temperament played a vulnerable role against the workers’ own occupational stress.

#### Anxious temperament and occupational stress

Anxious temperament has been associated with many traits: worry, vigilance, tension, oversensitivity, unrestful sleep, and gastrointestinal symptoms [22, 41]. Anxious temperament has also been documented as a high-risk factor for depressive symptoms [10], a robust predictor of anxiety and depressive disorders [16] and a strong predictor of suicide attempts [6]. A positive correlation between anxiety and perfectionism has been reported [42, 43].

In table 3, higher scores of anxious temperament are associated with higher job future ambiguity and lower support from coworkers. Workers with higher anxious temperament might feel higher job future ambiguity and lower support from coworkers. Sakai and colleagues reported that anxious temperament seemed to predispose individuals to the most prominent vulnerability to role conflict, role ambiguity, interpersonal conflict, and quantitative workload [30]. Our results were completely different, perhaps because temporary employees have been increasing as types of employment become more diversified, and “job future ambiguity” may since have become the most stressful factor for workers with anxious temperaments. Another explanation might be that workers with anxious temperament can cause their surrounding coworkers to tire of their worries or perfectionism, and therefore either objectively do not receive support from coworkers or subjectively perceive lower levels of support than expected. In sum, anxious temperament played a vulnerable role against the workers’ own occupational stress.

#### Depressive and cyclothymic temperaments, and occupational stress

Depressive temperament emerges as bound to routine, self-blaming, shy-nonassertive, sensitive to criticism, yet also self-denying, dependable and more inclined to work for someone else than be the boss [3]. Cyclothymic temperament is characterized as being rather tempestuous: labile with rapid shifts in mood; variable in sleep, energy, self-esteem and socialization; a dilettante and, perhaps by the same token, keen in perception and intense in emotions; and a romantic [3].

In this study, no relationship was found between occupational stress and depressive or cyclothymic temperament. The relationships between depressive temperament and change in workload, quantitative workload, job control [30], effort, and reward based on the Effort-Reward Imbalance (ERI) model [31, 32] have been reported previously. The relationships between cyclothymic temperament and role conflict, interpersonal conflict [30], effort/reward ratios based on ERI model [31, 32] have also been documented. Depressive temperament was found to be a hyper-adapted pattern to the workplace [32], and a gender difference was found such that women have higher scores on both depressive and cyclothymic temperament than do men [44]. These differences may actually be due to gender, but may also derive from differences in occupation or different methods for evaluating occupational stress.

### The effects of demographic variables on occupational stress

In the present study, after the model was adjusted for temperaments, gender was significantly related to role conflict, and position classification was significantly related to variance in workload and job control. Male managers more often reported conflicts with supervisors and lack of their support than did female managers, female managers reported lower levels of well-being than did male managers, and managers reported higher job demands than did non-managers of the same sex [45]. It is therefore necessary to pay attention to the effect of position, and not just factors such as gender and temperament, in surveys about work environment, occupational stress and the like.

Types of employment in Japan are very diverse, and lifelong employment is no longer universal. Therefore, we expected to discover a relationship between type of employment and occupational stress, but there was little association between the two. Future studies with many different kinds of occupations or different cultural backgrounds could reveal an effect of employment type.

### Limitations of this study

Several limitations in this study should be mentioned. First, the sample size was small, and only Japanese local government employees were surveyed. Second, occupational stress was evaluated by self-report; thus, the results may be influenced by response tendencies. Third, the moderate response rate for our survey questionnaire (84.3%) might have created selection bias. Fourth, it cannot be known with certainty from our data that the relationship between temperament and occupational stress is causal; nor can the direction of any such causality be established. For example, it is possible that job stressors affect scores on measures of temperament. This is unlikely, however, given the relative stability of temperament as compared with stress. A final limitation is that specific temperaments’ effects on career choice were not addressed in this study. Two previous studies have reported a relationship between temperament profile and professional choice among students and cadet officers [46, 47], and therefore workers with different temperaments might choose different occupations. Future study will need to focus on this point. Finally, although the TEMPS-A scores may be related to mental status (such as depressive symptoms), mental conditions were not evaluated in this study. None of the subjects in this study were unemployed, but employees working while having mental disorders might exist. Therefore future studies will need to take subthreshold mental disorders (such as depressive, anxiety symptoms) into consideration. In future research, a cohort or longitudinal design to address the relationship between temperament and the factors in the workplace would be beneficial.

### Temperaments in the workplace

Workers’ recognition of their own personal temperaments will contribute to self-care, and to the awareness of and relief from occupational stress. Workplace assessment and identification of temperaments, and recognition of these temperaments’ effects on occupational stress by workers themselves, and by coworkers and supervisors, will facilitate environmental coordination of individual temperaments.

Our results suggest specifically that workers with irritable and anxious temperaments should be identified because they may have higher-than-expected occupational stress. When subordinates with irritable temperament work in the same department, supervisors may be able to reduce these workers’ occupational stress by clarifying the nature of the work and paying more attention to large variances in workload and to intragroup conflict. For workers with anxious temperament, establishing and consolidating support from coworkers may lead to a reduction of occupational stress.

It would be helpful for organizational staff to provide supportive guidance on stress coping styles according to a cognitive-behavioral therapeutic approach that is based on a worker’s temperamental tendency. Job counseling specific to each temperamental tendency and consideration of temperaments when job descriptions are created would be useful steps for better adaptation to occupational stress.

## Conclusions

This study demonstrated that workers’ hyperthymic temperament played a protective role against occupational stress, and that irritable and anxious temperaments played a vulnerable role against occupational stress. It was newly found in this study that hyperthymic temperament played a protective role and that anxious temperament played a vulnerable role against job future ambiguity. Recognizing one’s own temperament and the role it plays in well-being would lead to self-insight, and the recognition of workers’ temperaments by their supervisors or coworkers would help promote social support in the workplace. In the future, we hope that temperaments will receive more focus within the study and practice of occupational safety and health.
